# Hypoglycemia-Induced Basal Ganglia Infarct: A Rare Case of Metformin Toxicity in a Hemodialysis Patient

**DOI:** 10.7759/cureus.32449

**Published:** 2022-12-12

**Authors:** Rovena Collins, Ariana R Tagliaferri, Gabrielle LoBue, William Meng, Mourad Ismail

**Affiliations:** 1 Internal Medicine, St. Joseph's Regional Medical Center, Paterson, USA; 2 Internal Medicine, St. George's University School of Medicine, Paterson, USA; 3 Pulmonary and Critical Care, St. Joseph's Regional Medical Center, Paterson, USA

**Keywords:** metformin in dialysis patients, ischemic stroke, metformin-associated lactic acidosis, medication toxicity, metformin side effects

## Abstract

Metformin is the preferred agent in type 2 diabetes due to its efficacy, safety profile, cardioprotective benefits, weight loss, and accessibility in the market. However, Metformin should be prescribed with caution in patients with renal dysfunction and is contraindicated in those with a glomerular filtration rate (GFR) of less than 30 mL/min. Though extremely rare, accumulation of metformin due to poor renal clearance can cause metformin toxicity and subsequently cause lactic acidosis and hypoglycemia. The incidence is estimated at less than 10 events per 100,000 patients. Hypoglycemia has been shown to induce ischemic strokes in previous case reports; however, only one other case control study has shown hypoglycemia-induced strokes in the setting of metformin toxicity. Herein, we present a rare case of hypoglycemia-induced ischemic stroke from metformin toxicity, in a patient undergoing maintenance hemodialysis. Our case report illustrates an extremely rare case of metformin toxicity that caused a hypoglycemic-induced ischemic infarct.

## Introduction

Metformin is a biguanide drug, which acts by decreasing the production of glucose in the liver, inhibiting the breakdown of glycogen, decreasing the intestinal absorption of glucose, and increasing insulin sensitivity [1-3]. Metformin is usually dosed from 500 to 2,550 milligrams (mg) per day and administered with meals to avoid gastrointestinal symptoms such as cramping and diarrhea. The half-life is about 20 hours, and the drug is renally excreted [1]. It is recommended that no more than 250 mg daily or 500 mg daily be used in patients undergoing peritoneal dialysis or hemodialysis, respectively [4]. Higher doses can lead to inadequate clearance, resulting in toxic accumulation of Metformin, causing a myriad of complications [3,4]. Other conditions that predispose patients to poor clearance and adverse reactions include cirrhosis, heart failure, and sepsis [5].

Metformin toxicity occurs in 1-10 per 100,000 individuals annually [5]. The symptoms of metformin-induced toxicity can range in severity and type, but often include nausea, diarrhea, vomiting, hypotension, and altered mental status [5]. Current literature has described several complications of this condition, including respiratory failure, severe acidosis, hypoglycemia, and even rarer adverse reactions such as DRESS syndrome and/or blindness [5,6]. The mortality due to complications, ranges from 17.2% to 50%, and thus a thorough clinical history should increase suspicion in patients presenting with these signs [5,6].

Hypoglycemia itself can induce a sequence of pathways that may induce ischemic stroke [7]. One mechanism is the activation of multiple platelets and cytokine pathways, leading to increased fibrinogen formation [7]. Recurrent hypoglycemic episodes also cause an increase in vascular adhesion molecules and cytokines leading to a cascade of inflammatory-based processes that cause atherogenesis and thrombotic complications [7]. Atherosclerosis is one of the leading risk factors associated with ischemic stroke, and thus it can be inferred that recurrent episodes of hypoglycemia can indirectly cause vascular complications, such as ischemic stroke [7].

## Case presentation

A 50-year-old female with a past medical history of end-stage renal disease (ESRD) on hemodialysis, diabetes mellitus, and hypertension presented to the Emergency Department (ED) with generalized abdominal pain, vomiting, and diarrhea for one day prior to admission. The patient reported hypoglycemic episodes for the past few weeks but was otherwise in her normal state of health. Of note, her diabetes mellitus was currently being treated with Metformin 1,000 mg twice daily and Insulin glargine 10 units, injected nightly. Of note, the patient had been on this dosage for the last three weeks prior to admission. The dose was recently increased due to poor glycemic control. Additionally, the patient was taking generic Metformin.

On arrival, the patient was hemodynamically stable (113/56 mmHg, 69 beats per minute) and afebrile; however, initial fingerstick glucose was 47 mg/dL. This blood pressure was consistent with her baseline on prior admissions. She was lethargic but initially protected her airway. The examination was otherwise remarkable for tenderness to superficial palpation of the right upper quadrant, with negative Murphy’s sign and no organomegaly. Labs were remarkable for arterial blood gas (ABG) pH 6.89, PCO_2_ 22 mmHg, PAO_2_ 87 mmHg, HCO_3 _- 4.2 mmol/L, lactic acid of 20 mmol/L, and plasma metformin level of 46 mcg/mL (reference range 1-2 mcg/mL). Serum chemistry was significant for sodium of 146 mEq/L, potassium of 4.7 mEq/L, bicarbonate of 3 mEq/L, the glucose of 35 mg/dL, BUN of 86 mg/dL, Creatinine of 10.22 mg/dL; however, troponin and liver function enzymes were within normal limits. A complete blood panel showed a leukocytosis (WBC 15x10^3/mm3) and left shift (segmented neutrophils 83x10^3/mm3) without bandemia. An electrocardiogram (EKG) showed sinus rhythm without any ST or T wave abnormalities. A computerized tomography (CT) of the abdomen with contrast revealed sub-segmental atelectasis at the lung bases, mild emphysematous changes, cardiomegaly, reflux esophagitis, and a distended gallbladder with a 3-mm calculus and perivesical fat stranding.

**Figure 1:**
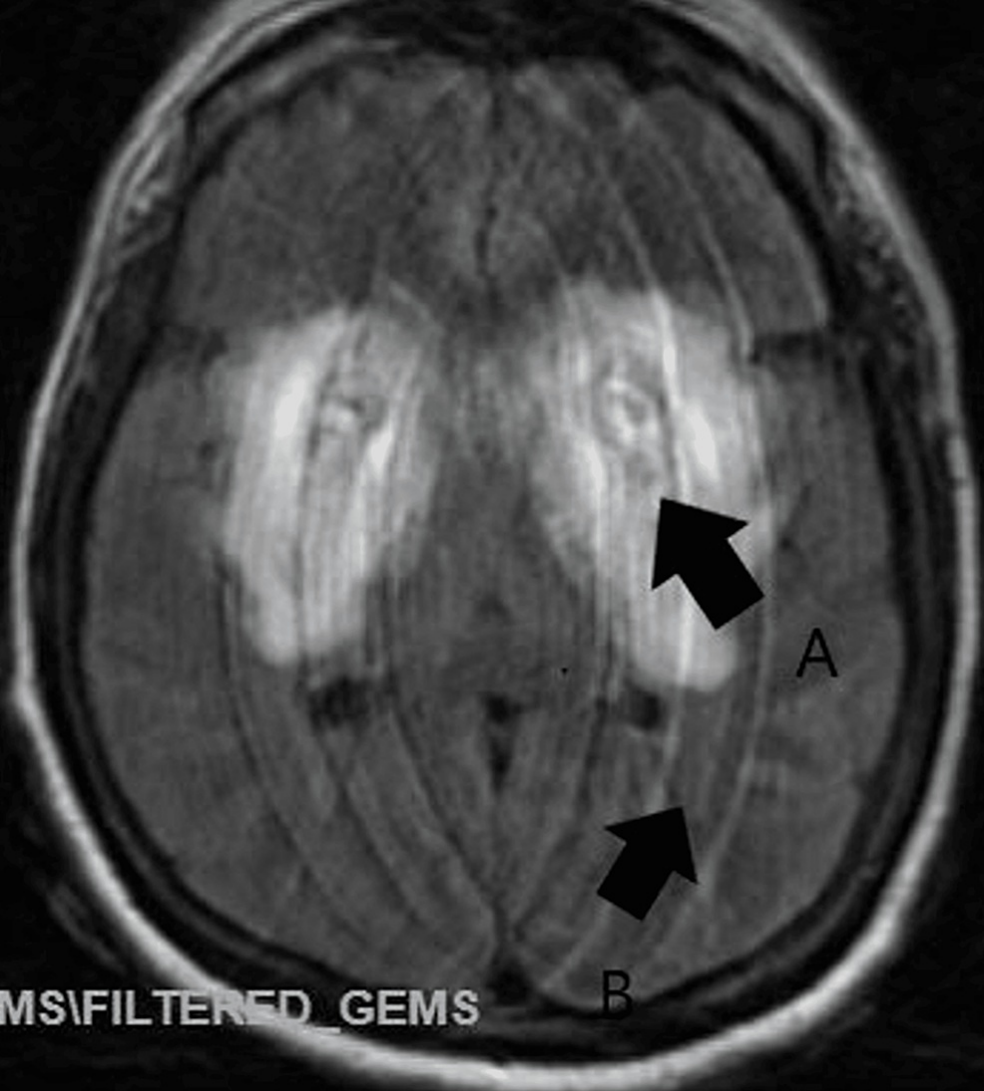
Initial MRI without contrast revealing concomitant infarction and hemorrhaging of the bilateral lentiform nuclei. A limited multiplanar/multisequence MRI of the brain was performed without contrast. The figure was obtained from FLAIR AX imaging. Limited exam due to motion degradation, demonstrating restricted diffusion (Arrow A) within bilateral lentiform nuclei suggestive of infarction as well as hemorrhage with associated marked edema (Arrow B) in the adjacent brain parenchyma suspicious for hypoxic-ischemic changes. No hydrocephalus was seen.

**Figure 2:**
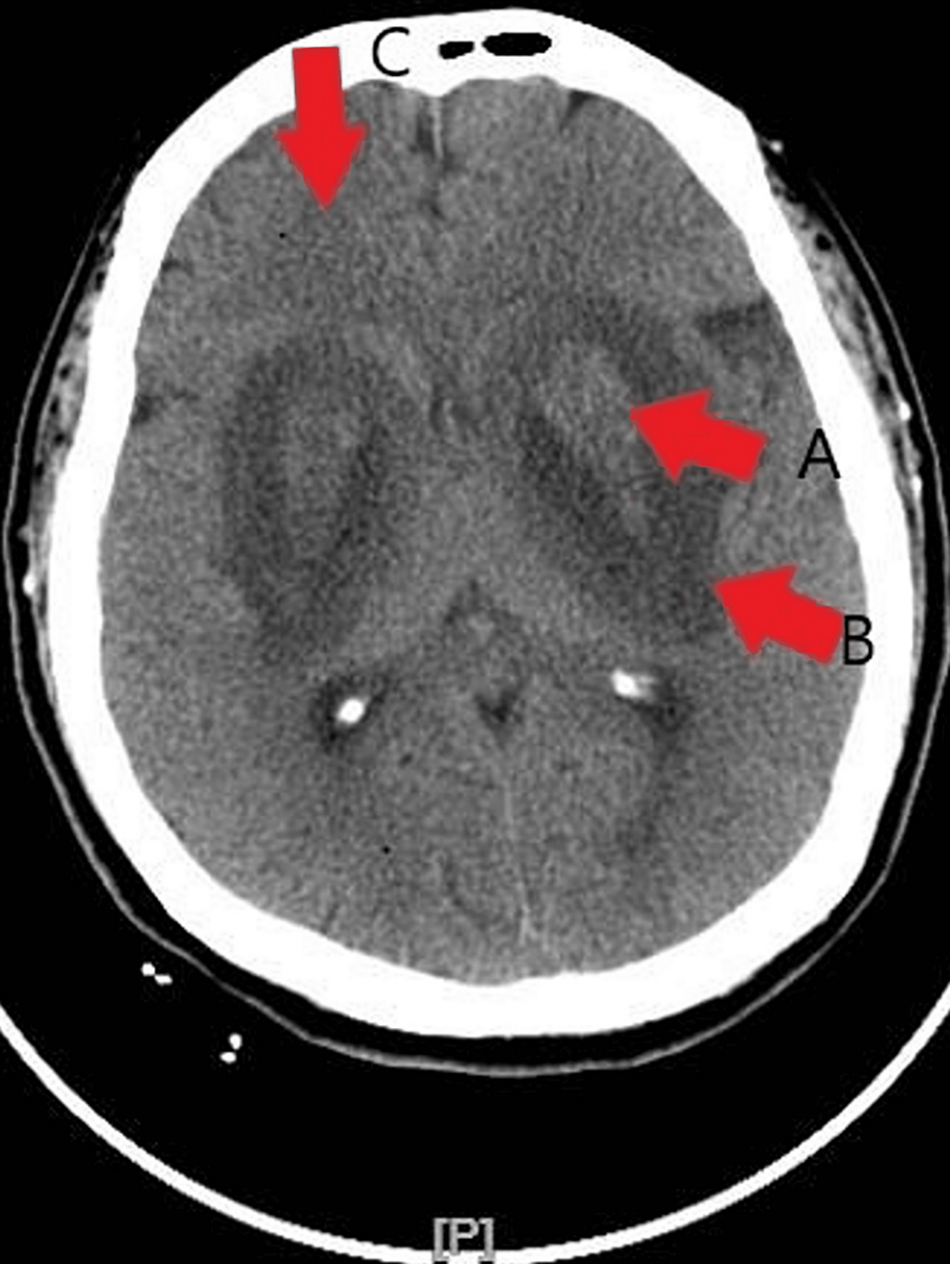
Initial CT head without contrast revealing basal ganglia infarct and possible lentiform nuclei hemorrhaging. Multiple contiguous axial images were obtained from the skull base to the vertex without the administration of intravenous contrast. Sagittal and coronal 2D reformations were performed. There are areas of lucency in the temporal lobes bilaterally involving the lentiform nuclei (Arrow B). There are patchy hyper-densities of the lentiform nuclei bilaterally suggesting hemorrhage (Arrow A). There is marked lucency surrounding the basal ganglia on both temporal lobes (Arrow B), left caudate nucleus (Arrow B), adjacent bilateral frontal (Arrow C) and parietal lobes (Arrow C) suggesting ischemia or anoxic changes. There is no extra-axial collection or midline shift. There is a mass effect on the lateral ventricles especially the frontal horns and temporal horns without hydrocephalus.

The patient was initiated on intravenous normal saline, received three ampules of bicarbonate, and was started empirically on Vancomycin 1 gram daily and Piperacillin-Sulbactam 3.375 mg daily. After her third liter of fluids, her systolic blood pressure was noted to be in the 80s and a central line was inserted in the subclavian vein. She was treated with norepinephrine; however, the patient became obtunded, with non-bloody, nonbilious emesis, and was subsequently intubated for airway protection. She was transferred to the intensive care unit (ICU). Due to severe lactic acidosis, the patient was initially placed on a bicarbonate drip and underwent emergent hemodialysis. On hospital day 3, a routine electroencephalogram (EEG) and CT head without contrast were obtained, which showed moderate to severe diffuse encephalopathy and areas of hypodensity in both lentiform nuclei suggestive of hemorrhage with marked lucency surrounding the basal ganglia suggestive of anoxic or ischemic changes (Figure 1). Neurology was consulted and a follow-up Magnetic resonance imaging (MRI) demonstrated restricted diffusion within the bilateral lentiform nuclei suggestive of infarction as well as hemorrhage with associated edema in the adjacent brain parenchyma (Figure 2). The patient was extubated, weaned off pressors, and transferred to the Neurology floor. On hospital day 10, a rapid response was called for emesis with lethargy and a Glasgow Coma Scale of 5, for which she was intubated and transferred back to the ICU for acute encephalopathy.

At this stage, it was unclear if this was due to neurological sequelae from the stroke, metabolic etiology, or sepsis. A repeat CT head without contrast demonstrated stable basal ganglia hyper-density with marked surrounding edema and a new mild midline shift to the right. Neurosurgery did not recommend intervention at this time. Four days later, the patient became hypotensive during hemodialysis after receiving her scheduled antihypertensives and upon repeat examination, the patient had anisocoria without reflexes and was unresponsive. Another CT of the head was performed, showing worsening edema and midline shift with near-complete effacement of the sulci and increased intracranial pressure. She was treated with head-of-bed elevation and hypertonic saline. The prognosis was discussed with the family and on the following day was declared brain dead.

## Discussion

Metformin toxicity typically presents with mainly gastrointestinal symptoms such as nausea, abdominal pain, and vomiting, however more severe symptoms such as lethargy, respiratory failure, and altered mentation can occur [5,6]. Rare side effects, such as blindness and DRESS syndrome have also been reported [5,6]. Metformin toxicity is associated with a high mortality rate if not diagnosed and treated early, as illustrated in our patient’s case [2,8]. A thorough history and medication reconciliation are thus critical in these patients [8]. While the definitive diagnosis of metformin toxicity is obtained by measuring the metformin level in the plasma, this test is often sent to an external laboratory and is not readily available, therefore is typically a clinical diagnosis made from initial presentation and history [8]. In our patient, metformin toxicity was clinically suspected and then confirmed with a plasma metformin level of 46 mcg/mL (reference range 1-2 mcg/mL) even after the patient had already received treatment.

Lactic acid plays numerous roles in the development of metformin toxicity, but generally involves inhibition of complex-1 of the mitochondrial respiratory chain resulting in pyruvate conversion to lactate [2,8-10]. It also increases the conversion of glucose to lactate in the small intestine [8]. Metformin levels greater than 5 mcg/mL are associated with lactic acidosis [10]. Metformin-induced lactic acidosis is correlated with specific laboratory findings, such as a mean blood pH below 7.0 typically not seen in other types of lactic acidosis such as septic shock, cardiogenic shock, or hemorrhagic shock [8,9]. As our patient was receiving an inappropriately high dose of Metformin on hemodialysis and had a low blood pH with lactic acidosis, our suspicion for metformin toxicity was increased even before obtaining the results of her plasma metformin levels. It is thus imperative for physicians to consider metformin toxicity in those who meet most or all of the following criteria: recent metformin use, elevated lactate levels >15 mmol per liter, an anion gap >20 mmol per liter, severe acidemia with a pH <7.1, a serum bicarbonate level <10 mmol per liter, and a history of renal insufficiency defined as estimated GFR <45 mL/min/1.73 or a serum creatinine level >2.0 mg per deciliter [8].

Though hypoglycemia has been reported in case studies as a consequence of toxic metformin levels, the correlation between metformin toxicity and strokes is very rare and was only described in one case-control study [1-3]. The study included 17,760 patients with diabetes mellitus on hemodialysis and found that the risk of both hemorrhagic and ischemic strokes was higher in metformin users [2]. Though metformin toxicity may not directly lead to hemorrhagic or ischemic stroke, it is associated with hypoglycemia which has been known to mitigate pathways leading to both ischemic and hemorrhagic strokes [7,10,11]. Preclinical studies have provided strong evidence that hypoglycemia may accelerate various proinflammatory and procoagulant mechanisms that increase the risk of cerebrovascular events [7,9,11]. Additionally, hypoglycemia has been associated with platelet activation as well as an increase in inflammatory markers such as IL-6, TNF-alpha, endothelin-1, and C-reactive protein, all of which leads to fibrinogen formation and contribute to the onset of macrovascular events such as ischemic stroke [7,10]. We can conclude that hypoglycemia played a significant role in the development of our patient’s strokes because she had a subjective history of recent and numerous hypoglycemic episodes at home and presented with critical hypoglycemia. It is plausible that multiple hypoglycemic events from toxic metformin levels triggered pro-inflammatory and pro-thrombotic pathways that inevitably resulted in our patient’s strokes and ultimately her demise.

## Conclusions

Herein, we discuss a case of metformin toxicity that caused a hypoglycemic-induced ischemic infarct. Though previous studies have shown a correlation between hypoglycemia and ischemic strokes, only one prospective study has shown this in the setting of metformin toxicity. Throughout the patient’s hospitalization, we witnessed all the complications from metformin toxicity including severe lactic acidosis, hypoglycemia, and cerebrovascular events. This case highlights the significance of appropriate metformin dosing in patients with renal dysfunction and/or need for dialysis, as well as the importance of close monitoring for common or less common complications.
